# *KCNB1* mutation impairs neuronal differentiation by disrupting gene expression temporal regulation and neuron-specific pathways

**DOI:** 10.3389/fneur.2026.1739214

**Published:** 2026-01-28

**Authors:** Yufan Guo, Lifang Wu, Danfeng Ye, Xueting Lin, Yuting Jin, Chudi Zhang, Yuting Lou, Pu Miao, Ye Wang, Bijun Zhang, Jianhua Feng

**Affiliations:** 1 Department of Pediatrics, The Second Affiliated Hospital of Zhejiang University School of Medicine, Hangzhou, Zhejiang, China; 2 Department of Pediatrics, Suichang County People's Hospital, Lishui, China; 3 Department of Pediatrics, Songyang County People's Hospital, Lishui, China

**Keywords:** iPSC, *KCNB1*, neuronal differentiation, RNA-seq, synapse-related pathways

## Abstract

**Introduction:**

This study aims to rigorously evaluate the consistency and reliability of a pluripotent stem cell (PSC) differentiation system and explore how the *KCNB1* mutation disrupts the temporal regulation of gene expression during neuronal differentiation and modulates neuron function-related pathways.

**Methods:**

Induced pluripotent stem cells (iPSCs) derived from a patient carrying a *KCNB1* variant (c.990G > T, p.Glu330Asp) and from a healthy donor were differentiated into neurons. Differentiation and RNA expression were assessed at multiple time points. Immunofluorescence, RNA sequencing, fuzzy c-means clustering, and pathway analyses were performed.

**Results:**

The differentiation system was successfully established, with cells exhibiting stage-appropriate morphology and maturing into neurons. RNA sequencing revealed consistent gene expression patterns at the neural progenitor cell (NPC) stage but significant differences at the neuron stage between the *KCNB1* mutant patient and the healthy donor. Notably, *KCNB1* expression was lower in the patient’s neurons. Genes specifically clustered in healthy neurons were enriched in synapse-related pathways, while genes clustered in patient neurons were associated primarily with basic cellular metabolism pathways and abolished neuron-specific pathways.

**Conclusion:**

Low expression of *KCNB1* disrupts the temporal pattern of gene expression and related neuron-specific pathways during neuronal differentiation and impairs neuronal differentiation and maturity.

## Introduction

Early-onset epileptic encephalopathies (EOEEs) are severe neurodevelopmental disorders that primarily emerge in infants and young children. They are characterized by frequent epileptic seizures, abnormal electroencephalogram (EEG) patterns, and progressive decline in cognitive and motor functions, imposing a heavy burden on patients, their families, and society (1, 2). Current clinical management mainly focuses on symptomatic seizure control, while treatments targeting the underlying molecular pathological mechanisms are still limited (2, 3). One significant obstacle to advancing these treatments is the inaccessibility of human neural tissue, which hinders the detailed investigation of the molecular mechanisms influencing the onset and progression of EOEEs. With advances in molecular genetics technologies, such as whole-exome sequencing, numerous genes associated with EOEEs have been identified, including *CSMD1* (4), *FBRSL1* (5), *GABRA1* (6), *TANC2* (7), *SCN1A* (8), *SRCAP* (9), *SLC2A1* (10), *SZT2* (11), *LAMA5* (12), and *ZFHX3* (13). Among these causative genes, ion channels represent a well-established functional mechanism in the pathogenesis of epilepsy (14–16).

*KCNB1*, which encodes the voltage-gated potassium channel Kv2.1, has attracted increasing attention in EOEE research (17, 18). The Kv2.1 channel is widely expressed in the neurons of the central nervous system (CNS) and plays a critical role in action potential repolarization (19). Since the first report of a *KCNB1* variant in EOEEs in 2012, more than 100 pathogenic or likely pathogenic variants have been listed in the ClinVar database.^1^ These variants disrupt the structure and function of KCNB1 by reducing channel current density, altering voltage-dependent activation/inactivation properties, or impairing membrane localization, which ultimately leads to dysregulation of neuronal electrophysiological function (20). However, existing models (such as mouse models or cell lines) fail to fully recapitulate human neural development and pathological features due to interspecies differences or the lack of authentic neuronal differentiation (21, 22).

Induced pluripotent stem cell (iPSC) technology provides a tool for studying human neuronal development. Derived from patient blood cells or fibroblasts, iPSCs possess unlimited self-renewal capacity and pluripotency, enabling their differentiation into various functional neural cell types, such as neurons, astrocytes, and other neural cells (23–25). iPSCs can retain the patient’s genetic background, recapitulate neuronal development, and serve as a scalable platform for drug screening, making them an increasingly valuable model in neurological research. Recent studies have utilized iPSCs to investigate the impact of mutations on neuronal development and function (26–28), with a growing focus on how *KCNB1* mutations affect neurodevelopment and epilepsy (29–31). However, key mechanisms, such as *KCNB1*’s interaction with other pathogenic genes and its dynamic expression pattern during neuron differentiation and development, are still unresolved.

In this study, we used *KCNB1*-mutant iPSCs derived from a 6-year-old female patient carrying the *KCNB1* variant (c.990G > T, p.Glu330Asp) as a research model to systematically characterize dynamic changes in cell morphology and gene expression during their induced differentiation into neurons.

## Methods

### Induced differentiation of iPSCs

The iPSC samples and methodology were established in our previous study. *KCNB1*-mutant iPSCs were obtained from a 6-year-old female patient carrying the *KCNB1* variant (c.990G > T, p.Glu330Asp), who was diagnosed with early infantile epileptic encephalopathy 26 (EE26) (26), while healthy iPSCs were derived from a healthy donor. Detailed reagents and materials are listed in Table 1. For forebrain neuron culture, culture plates were initially coated with 50 μg/mL poly-D-lysine (PDL) for 2 h and washed twice with Dulbecco’s phosphate-buffered saline (DPBS). After drying, the plates were then coated with 1 μg/mL laminin for 2 h.

**Table 1:** Reagents and antibodies.

Reagents and antibodies	Manufacturer	Accession number
DMEM F12	Gibco	11,320,033
Neurobasal™ medium	Gibco	21,103,049
B27 supplement (50X)	Gibco	17,504,044
N2 supplement (100X)	Gibco	17,502,001
L-glutamine (200 nM)	Gibco	25,030,081
Retinoic acid	MCE	HY-14649
SB431542	Selleck	S1067
LDN-193189 HCl	Selleck	S7507
Collagenase Type IV	Sigma-Aldrich	C5138
TrypLE™ Express (1X), Phenol Red	Gibco	12,605,010
Matrigel	BD	354,230
Recombinant Human/Murine/Rat BDNF	Novoprotein	C076
Recombinant Human GDNF Protein	Novoprotein	C226
Y-27632	Selleck	S6390
Laminin	Sigma-Aldrich	L4544
PDL	Sigma-Aldrich	P7886
L-Ascorbic acid	Sigma-Aldrich	A4403
DAPI	Sigma-Aldrich	28,718-90-3
MAP2	Abcam	ab11267
NeuN	Abcam	EPR12763

### Immunofluorescence

Cells were fixed with 4% formaldehyde in PBS for 15 min at neuron day 10, followed by treatment with PBS (0.3% Triton X-100 added) for 30 min. The cells were then blocked with 5% BSA for 30 min and incubated overnight at 4 °C with primary antibodies (Abcam, mouse anti-MAP2 1:100; Abcam, Rabbit anti-NeuN 1:100). After washing, the cells were incubated with the secondary antibody (Gibco, mouse 488 1:500) and DAPI for 1 h. Fluorescence images were captured using a microscope (Nikon TS2-FL). Detailed reagents and antibodies are listed in Table 1. The numbers of DAPI+, NeuN+, and MAP2 + cells were quantified from three independent differentiation experiments using Fiji-ImageJ (1.54p). Image acquisition and analysis were performed by an investigator blinded to sample identity (healthy donor vs. patient). A *t-*test was used for data analysis, and a *p*-value of < 0.05 was considered statistically significant.

### RNA extraction and sequencing

Total RNA was extracted using TRIzol (AM9738, Invitrogen) according to the manufacturer’s protocol. RNA concentration and purity were measured using NanoDrop (Thermo Fisher Scientific) and Agilent 2100 Bioanalyzer. Samples with an RNA integrity number (RIN) ≥ 7.0 were used for subsequent library preparation. mRNA was enriched using oligo(dT) magnetic beads, and the purified mRNA was fragmented into ~200 bp pieces. The quality of the libraries was assessed using Agilent 2,100 Bioanalyzer and quantified by qPCR. Finally, the libraries were sequenced on the Illumina platform (NovaSeq 6000) using a paired-end sequencing strategy (150 bp read length).

### RNA analysis

After adapter trimming and quality control using Trimmomatic,^2^ clean reads were aligned to the human reference genome obtained from the Ensembl database (http://ftp.ensembl.org/pub/release-104/fasta/homo_sapiens/dna). Then, STAR (v2.7.10a, https://github.com/alexdobin/STAR) was utilized for both reference genome building and clean data alignment. FeatureCount was used to count the number of reads for each gene. Differential expression analysis was performed with DESeq2 (v1.16.1). Genes with an adjusted *p*-value < 0.05 and |log2(fold change)| > 1 were considered significantly changed.

### Fuzzy C-means clustering

Genes from the healthy donor and the patient were grouped into different clusters using the fuzzy c-means algorithm in the Mfuzz (v.2.56.0) package (32).

### Visualization

Violin and line plots were generated using ggviolin and ggline in the ggpubr package (v.0.6.0, https://rpkgs.datanovia.com/ggpubr/), and the correlation plot among the samples was visualized using corrplot (v.0.92, https://github.com/taiyun/corrplot). Principal component analysis (PCA) was performed and visualized using ggfortify (v.0.4.17, https://github.com/sinhrks/ggfortify), FactoMineR (v.2.1.1, https://github.com/husson/FactoMineR), and factoextra (v.1.0.7, https://github.com/kassambara/factoextra). Gene Ontology (GO) and Kyoto Encyclopedia of Genes and Genomes (KEGG) enrichment analyses were performed and visualized using clusterProfiler (v.4.10.0, https://github.com/YuLab-SMU/clusterProfiler).

## Results

### Consistency and reliability of the induced differentiation system

**Figure 1:**
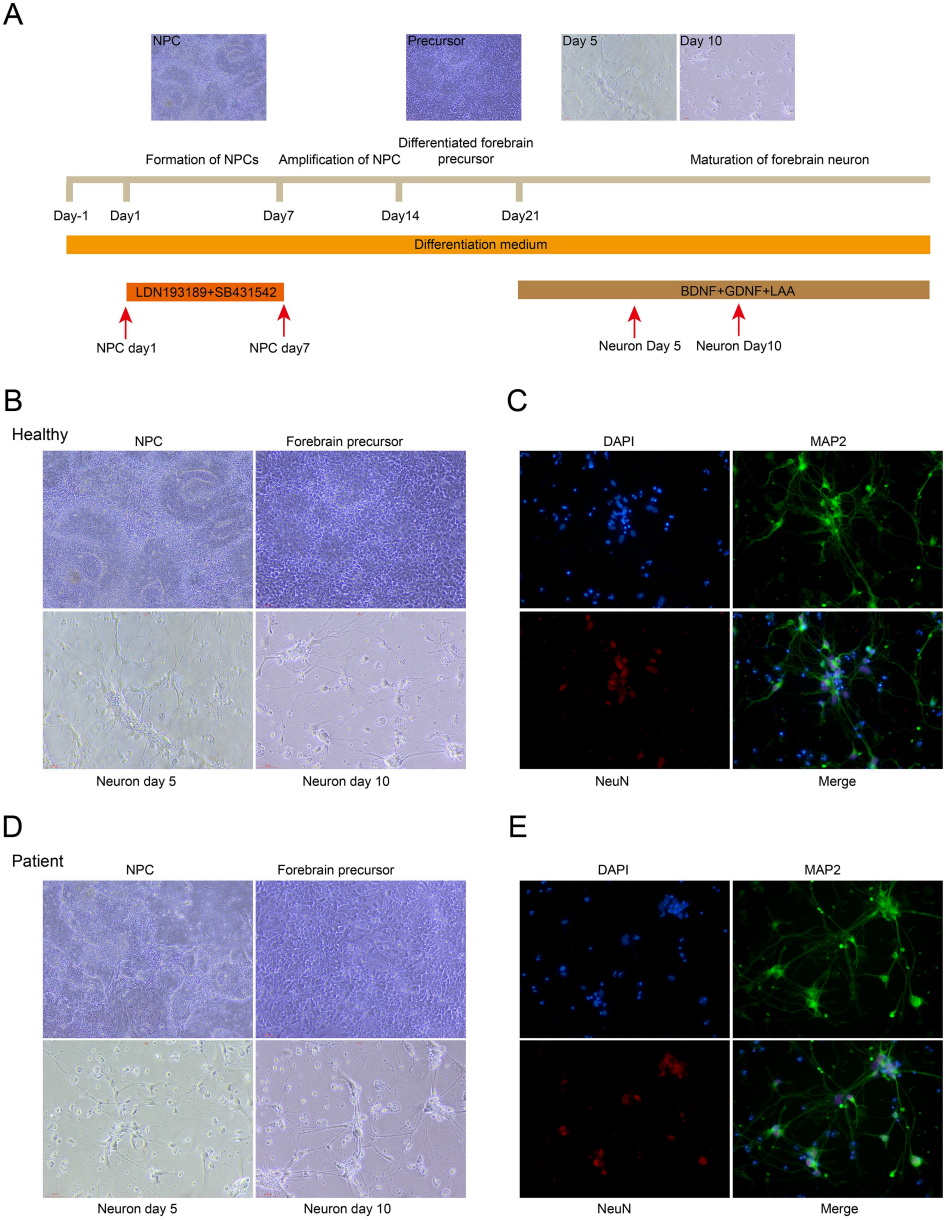
Induced differentiation of iPSCs. **(A)** Experimental schematic of the iPSC differentiation process. **(B)** Representative morphological images of healthy donor-derived NPCs at neuron day 5 and forebrain precursors at neuron day 10. **(C)** Representative images of immunofluorescence-stained neurons derived from the healthy donor. Neurons were stained with MAP2 (green) and NeuN (red). Nuclei were stained with DAPI (blue). Scale bar: 10 μm. **(D)** Representative morphological images of patient-derived NPCs at neuron day 5 and forebrain precursors at neuron day 10. **(E)** Representative images of immunofluorescence-stained neurons derived from the patient. Neurons were stained with MAP2 (green) and NeuN (red). Nuclei were stained with DAPI (blue). Scale bar: 10 μm. BDNF, Brain-Derived Neurotrophic Factor; GDNF, Glial Cell Line-Derived Neurotrophic Factor; LAA, L-Ascorbic acid.

The bone morphogenetic protein (BMP) inhibitor LDN193189 and the selective transforming growth factor-*β* (TGF-β) type I receptor inhibitor SB431542 were utilized to induce cell differentiation. Portions of the cell populations were collected at neural progenitor cell (NPC) day 1 and day 7. After NPCs were expanded and differentiated into forebrain precursors, the cells were treated with brain-derived neurotrophic factor (BDNF), glial cell line-derived neurotrophic factor (GDNF), and L-ascorbic acid (LAA). The samples were harvested at neuron day 5 and day 10 (Figure 1A).

To verify the reliability of the induced differentiation system, we performed immunofluorescence staining on healthy donor- and patient-derived (c.990G > T, p.Glu330Asp) NPCs, forebrain precursors, and cells at neuron day 5 and neuron day 10. NPCs exhibited characteristic spindle-shaped or polygonal morphology, with plump cell bodies and large, round nuclei. Forebrain precursor cells showed transitional morphology between NPCs and mature neurons. Cells at neuron day 5 showed typical morphological features of early-stage neurons, while by day 10, they exhibited characteristics of mature neurons, such as rounded or oval cell bodies and small, dense nuclei (Figures 1B,D).

Immunofluorescence staining was performed at neuron day 10 to assess neuronal maturation. Nuclei were stained with DAPI (blue); neuronal identity and cytoarchitecture were assessed using anti-MAP2 (green); and mature neuronal nuclei were labeled with anti-NeuN (red). The DAPI staining results showed the nuclear integrity of neurons, while the MAP2- and NeuN-positive staining indicated well-developed neuronal processes and a mature nuclear phenotype, respectively. Immunofluorescence showed that nearly all DAPI-stained cells were positive for NeuN (Figures 1C,E). Quantitative analysis revealed that 83.02% of patient-derived neurons and 87.19% of healthy donor-derived neurons were double-positive for NeuN and MAP2, with no significant difference between the two groups (Supplementary Figure S1). These results confirm that the neurons had achieved morphological and functional maturity by day 10.

### *KCNB1* mutation disrupts the temporal regulation of gene expression during neuronal differentiation

**Figure 2:**
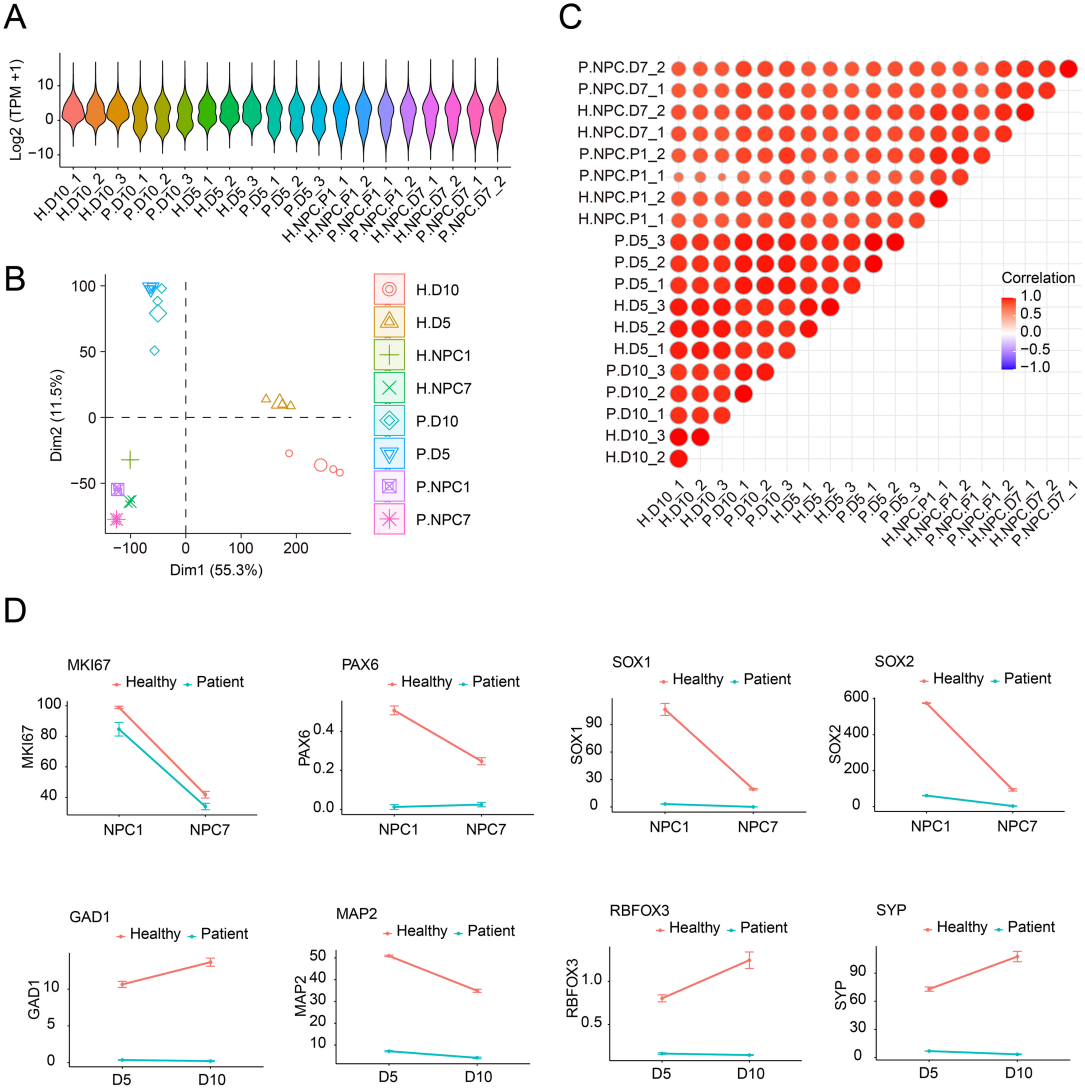
RNA sequencing identified abnormal marker genes. **(A)** Violin plots showing gene expression distributions across all samples. Each sample is indicated by a distinct color. **(B)** PCA of RNA-seq data. The PCA plot was generated using the first two principal components (PC1 and PC2), with each dot representing an individual group. Different colors and shapes denote samples from distinct experimental groups. Sample clustering within the same group and separation between different groups reflect the consistency of biological replicates and the transcriptomic differences induced by experimental treatments, respectively. **(C)** Correlation plots across all samples based on TPM values (transcripts per million). The color intensity in each cell represents the magnitude of the correlation coefficient: Warmer colors (red) indicate a higher positive correlation (closer to 1), while cooler colors (blue) indicate a lower correlation (closer to 0). **(D)** Expression changes of classical marker genes across NPC and neuronal stages. Error bars represent the SEM. TPM: transcripts per million; H.D.10: healthy neuron day 10; P.D.10: patient neuron day 10; H.D.5: healthy neuron day 5; P.D.5: patient neuron day 5; H.NPC.P1: healthy NPC day 1; P.NPC.P1: patient NPC day 1; H.NPC.P7: healthy NPC day 7; P.NPC.P7: patient NPC day 7.

To investigate the dynamic changes during differentiation, RNA sequencing was performed on cells from both patient and healthy donor lines at four stages: NPC day 1, NPC day 7, neuron day 5, and neuron day 10. We initially characterized the expression levels of all genes and found that gene expression distributions were relatively consistent across all samples, indicating the absence of abnormal specimens or data (Figure 2A). PCA showed that the healthy donor- and patient-derived samples clustered closely at NPC time points. In contrast, during neuronal stages (day 5 and day 10), the healthy donor-derived samples formed a distinct cluster separate from the patient-derived samples (Figure 2B), suggesting divergence in gene expression programs upon neuronal maturation. Correlation analysis between the groups showed low consistency between the NPC and neuron samples (Figure 2C). We further characterized the expression of marker genes at different differentiation stages. The proliferation marker (MKI67) was lower at NPC day 7 compared to NPC day 1 in both groups, while key early neural markers (PAX6, SOX1, and SOX2) demonstrated a significant decrease in healthy donor-derived cells but remained at low levels in patient-derived cells across both NPC time points. In contrast, mature neuronal markers (*GAD1*, *RBFOX3*, and *SYP*) showed a significant increase in healthy donor-derived cells but remained markedly lower in patient-derived cells. Although MAP2 expression decreased slightly at day 10 in healthy donor-derived cells, it remained markedly higher than in patient-derived neurons (Figure 2D). These findings indicate that KCNB1 mutations may contribute to gene dysregulation in patient cells, ultimately affecting the terminal state and function of neurons during differentiation.

### Analysis of stage-specific developmental genes

**Figure 3:**
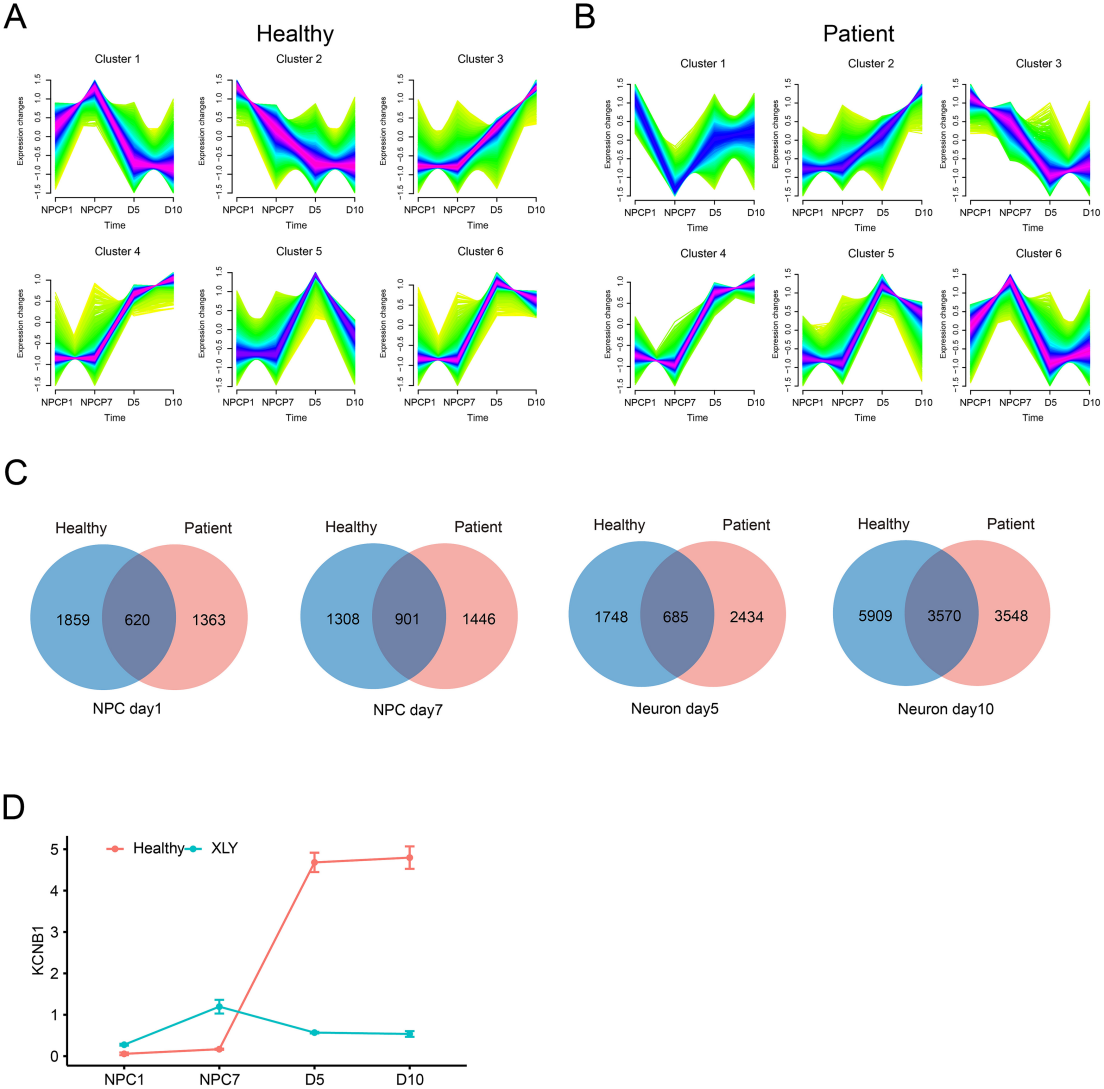
Analysis of stage-specific developmental genes. **(A)** Fuzzy *c*-means clustering identified six distinct temporal patterns of gene expression in healthy donor-derived cells. The *x*-axis represents the four developmental stages, while the *y*-axis shows log2-transformed, normalized intensity ratios at each stage. **(B)** Fuzzy *c*-means clustering identified six distinct temporal patterns of gene expression in patient-derived cells. The *x*-axis represents the four developmental stages, while the *y*-axis shows log_2_-transformed, normalized intensity ratios at each stage. **(C)** Overlap of stage-specific genes between healthy donor- and patient-derived cells. **(D)** *KCNB1* expression changes across developmental stages in healthy donor- and patient-derived cells.

We applied the fuzzy c-means algorithm to cluster gene expression profiles across all developmental stages. Six unique expression clusters were identified in both healthy donor- and patient-derived samples, representing groups of genes under different regulatory control during development (Figure 3A). In the healthy donor, Cluster 2 consisted of downregulated genes, while Cluster 1 consisted of upregulated genes. Clusters 1, 5, and 6 showed stage-specific high expression at NPC day 7 and neuron day 5 (Figure 3A). In the patient-derived samples, Cluster 3 consisted of downregulated genes, while Cluster 2 consisted of upregulated genes, and Clusters 1, 6, and 5 displayed stage-specific high expression at NPC day 1, NPC day 7, and neuron day 5, respectively (Figure 3B).

To compare stage-specific gene sets between the groups, we extracted the corresponding genes from the clusters at each time point. The number of stage-specific genes ranged from 1,308 to 5,909 in the two groups (Figure 3C). Notably, although *KCNB1* expression was similarly low in both groups during the NPC stage, it was strongly upregulated in healthy donor cells during neuronal maturation but remained markedly low in patient-derived cells (Figure 3D). These findings suggest that deficient *KCNB1* expression likely contributes to the altered gene expression patterns of mature neurons.

### *KCNB1* mutation abolishes neuron function-related pathways

**Figure 4:**
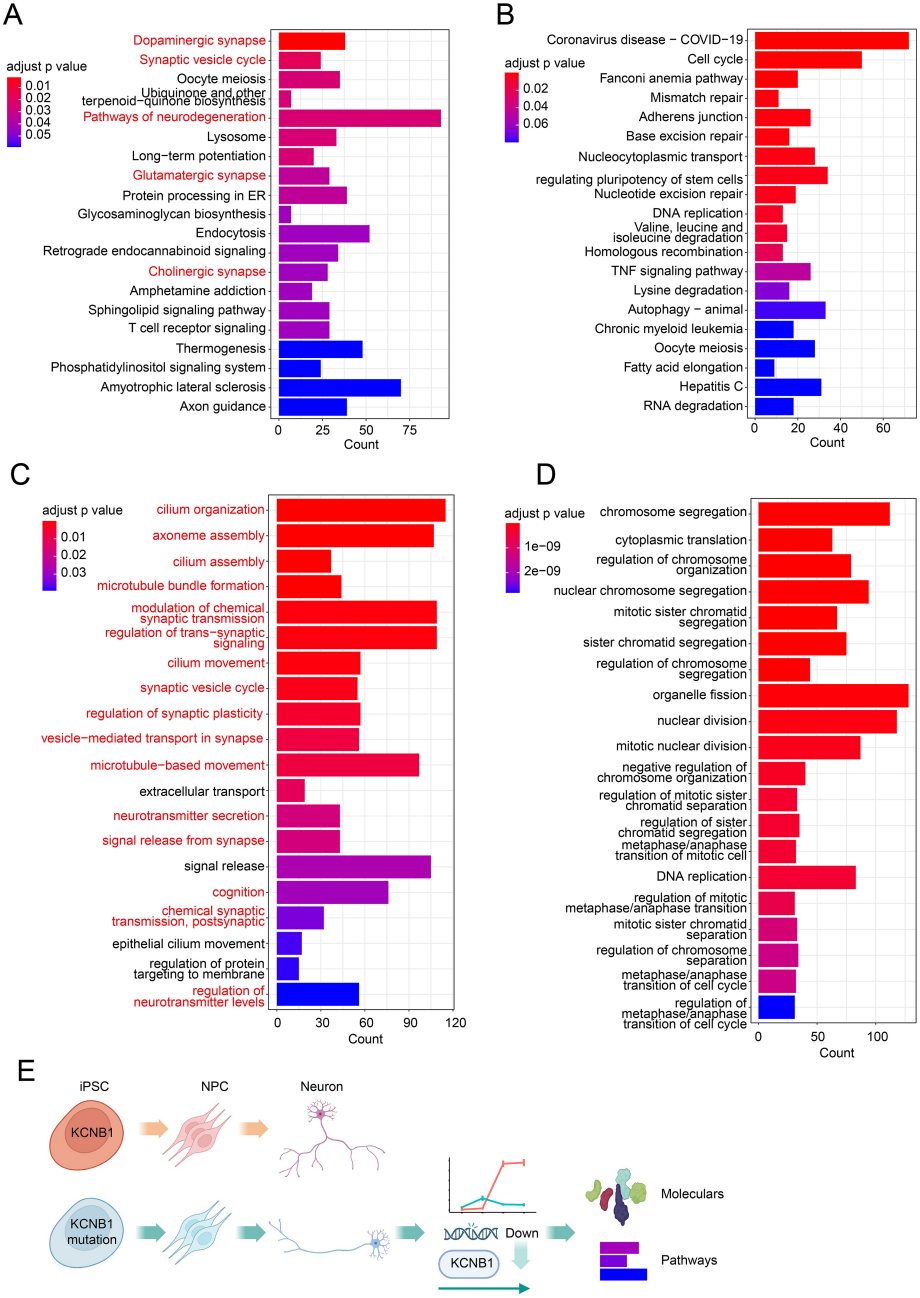
Pathway analysis revealed the absence of neuronal development pathways in patient- and healthy donor-derived cells. **(A)** KEGG pathway enrichment of healthy donor-specific genes at neuron day 10. **(B)** KEGG pathway enrichment of patient-specific genes at neuron day 10. **(C)** GO term enrichment of healthy donor-specific genes at neuron day 10. **(D)** GO term enrichment of patient-specific genes at neuron day 10. **(E)** Schematic diagram illustrating neural cell developmental defects caused by the *KCNB1* mutation.

To assess the potential impact of *KCNB1* on the gene expression patterns of mature neurons, we performed KEGG pathway and GO enrichment analyses on genes specifically expressed in healthy donor- and patient-derived samples at neuron day 10. KEGG analysis of healthy donor-specific genes revealed significant enrichment in synaptic pathways, including dopaminergic synapse, synaptic vesicle cycle, glutamatergic synapse, and cholinergic synapse (Figure 4A). In contrast, pathways related to neurons and neurotransmitters were absent in patient-derived cells (Figure 4B). GO analysis was consistent with the KEGG findings. Terms associated with neuronal development and function were highly enriched in healthy donor-derived cells, such as synapse, cilium, and microtubule-associated pathways (e.g., cilium organization/assembly/movement and microtubule bundle formation/movement), as well as processes involved in synaptic function, such as modulation of chemical synaptic transmission, regulation of trans-synaptic signaling, synaptic vesicle cycle, regulation of synaptic plasticity, synaptic transport, neurotransmitter secretion, and signal release from synapse (Figure 4C). In contrast, patient-specific genes were primarily associated with basic cellular metabolic processes, with no enrichment observed for neuron-specific functions (Figure 4D). Collectively, these results indicate that *KCNB1* deficiency disrupts dynamic changes in gene expression and molecular pathways, ultimately leading to the abolition of synaptic and neuronal pathways essential for mature neuron function (Figure 4E).

## Discussion

This study systematically investigated the impact of the *KCNB1* mutation (c.990G > T, p.Glu330Asp) on neuronal differentiation using patient-derived iPSCs, integrating morphological observations, immunofluorescence staining, RNA sequencing, and pathway analyses. The results show that reduced *KCNB1* expression leads to dysregulation of gene expression patterns and loss of functional molecular pathways in patient neurons, providing insight into potential molecular mechanisms underlying *KCNB1*-associated early-onset epileptic encephalopathies (EOEEs).

### Consistency and reliability of the induced differentiation system

A rigorous differentiation pipeline was established in this study. iPSCs were induced into neural progenitor cells (NPCs) using the BMP inhibitor LDN193189 and the TGF-*β* type I receptor inhibitor SB431542 and then matured into forebrain-derived neurons with BDNF, GDNF, and LAA. Morphological dynamic tracking demonstrated that the differentiation process was consistent with natural neural development and previous reports of iPSC-derived neuronal differentiation.

Furthermore, immunofluorescence staining at neuron day 10 confirmed the functional maturity of differentiated cell populations. The co-expression of MAP2 (dendritic marker) and NeuN (mature neuron marker) in DAPI-positive cells confirmed that >80% of cells were mature neurons with intact soma–dendrite structures, validating the reliability of the differentiation pipeline.

### *KCNB1* mutation disrupts gene expression during neuronal differentiation

RNA sequencing analysis revealed critical “stage-dependent divergence” in gene expression between *KCNB1*-mutant and healthy cells. During the NPC stages (day 1 and day 7), principal component analysis (PCA) showed overlapping distributions, with consistent expression of proliferation-related markers (e.g., MKI67) and NPC-specific markers (PAX6, SOX1, SOX2) in both healthy donor- and patient-derived samples (Figures 2B,D) (33–36). These findings suggest that the *KCNB1* mutation does not substantially influence the early neural induction of iPSCs.

However, a clear difference was observed at the neuronal stages (neuron day 5 and day 10). Cells from the healthy group upregulated neuron-specific markers [e.g., *GAD1* for GABAergic neurons, *RBFOX3* for mature neurons, and *SYP* for synaptic vesicles (37–39)] (37–39) while downregulating NPC markers. In contrast, these neuronal genes remained low in the *KCNB1*-mutant sample (Figure 2D). *KCNB1* expression was low in both groups at the NPC stage, but it was significantly upregulated in healthy neurons (day 5, day 10); however, it retained low levels in mutant cells at all time points. These results suggest that *KCNB1* functions as a “late-stage regulator” of neuronal differentiation, influencing the transition from NPCs to functional neurons.

### *KCNB1* mutation abolishes neuron function-related pathways

KEGG and GO analyses at neuron day 10 revealed the functional consequences of *KCNB1* deficiency. Genes clustered in healthy donor-derived cells were enriched for synaptic functions (e.g., dopaminergic/glutamatergic/cholinergic synapses and synaptic vesicle cycle) and neuronal development (e.g., cilium organization, microtubule bundle formation, and neurotransmitter secretion). These pathways are associated with neural excitability, synaptic plasticity, and circuit formation. In contrast, genes from *KCNB1-*mutant cells were primarily enriched in basic cellular metabolism pathways, with no neuron-related pathway enrichment. Similarly, Forzisi-Kathera-Ibarra et al. (40) revealed dysregulation of synapse organization and neurotransmitter signaling, as well as metabolic abnormalities, in KCNB1-null mice compare to wild-type mice. These findings further confirm that *KCNB1* plays a crucial role in synaptic function and neuronal development.

Although *KCNB1* mRNA expression remained low in patient-derived neurons (Figure 3D), morphological development was largely intact, and the proportion of mature neurons co-labeled with MAP2 and NeuN showed no significant difference compared to controls (Supplementary Figure S1). This suggests that the loss of neuronal function-related pathways caused by the p.Glu330Asp variant is not primarily driven by selective survival of immature neurons or widespread degeneration, as observed with some mutations (29), but it is more likely due to the mutation specifically disrupting the gene expression program essential for late-stage neuronal differentiation.

The underlying mechanisms may be associated with two aspects. First, *KCNB1* directly regulates membrane potential and calcium homeostasis in mature neurons, and its deficiency may disrupt calcium-dependent signaling cascades (e.g., Ca^2+^/CREB molecular pathways), thereby affecting the expression of synaptic and developmental genes. Second, the p.Glu330Asp variant may impair the non-conducting functions of the Kv2.1 channel, disrupting the signal transduction of protein complexes in which it participates, leading to abnormal cascades from synaptogenesis to gene transcription. Kv2.1 regulates cortical neuronal development and epileptogenesis by forming complexes with cell adhesion molecules, such as integrins (29, 41, 42). A recent study further showed that the p.Arg312His variant in the *KCNB1* gene primarily impairs the non-conducting signaling function of IKCs, leading to severe neurodevelopmental phenotypes, including neuronal migration disorders, abnormal cortical stratification, reduced synaptic connections, and epileptic behaviors (42). In addition, Kv2.1 interacts with scaffolding proteins (e.g., syntaxin 1A) to modulate synaptic vesicle release (43); the mutation may indirectly impair the assembly of synaptic complexes, downregulating synaptic pathway genes via feedback inhibition. In our study, the loss of pathways related to synaptic function and neuronal development, coupled with enrichment in basic cellular metabolism in patient-derived neurons, suggests that the p.Glu330Asp variant likely impairs synaptic function and neural development primarily by affecting non-ionic signaling rather than ion channel conductance. These findings provide a novel perspective for understanding the pathogenesis of *KCNB1*-related encephalopathy.

## Conclusion

Our study demonstrates that *KCNB1* deficiency impairs the late-stage maturation of neurons by disrupting the expression of neuronal markers, dysregulating numerous genes, and abolishing synapse-related molecular pathways. These findings highlight the key role of *KCNB1* in neuronal development and synaptic function and provide a new perspective for understanding the pathogenesis of *KCNB1*-related EOEEs.

### Limitations

Several limitations of this study should be acknowledged. The study used iPSCs derived from a single patient carrying the *KCNB1* mutation and a single healthy donor. The absence of multiple independent patient lines limits the generalizability of the findings across individuals with different genetic backgrounds or variant types. Furthermore, although isogenic controls (e.g., gene-corrected patient lines) are considered the gold standard for controlling genetic variability, they were not employed in this study. Relying on a single patient–control pair may introduce confounding effects from genetic background differences, which could influence the observed transcriptional and phenotypic outcomes. Finally, the conclusions of this study are based on transcriptomic data. Although we identified significant changes in the expression of key neuronal genes, such as *KCNB1*, *GAD1*, *RBFOX3*, and *SYP*, these results were not validated at the protein level or by transcriptional assays such as RT-qPCR. Future studies that incorporate multiple patient-derived lines, isogenic controls, and multi-omics validation (e.g., proteomics and electrophysiology) are essential to confirm and extend these mechanistic insights.

## Data Availability

The datasets presented in this study can be found in online repositories. The names of the repository/repositories and accession number(s) can be found in the article/Supplementary material.

## Footnotes

1
https://www.ncbi.nlm.nih.gov/clinvar

2
https://github.com/usadellab/Trimmomatic
