# Supplementary figures and images for: KCNB1 mutation impairs neuronal differentiation by disrupting gene expression temporal regulation and neuron-specific pathways
Source: Front Neurol. 2026 Jan 28;17:1739214. doi: 10.3389/fneur.2026.1739214 (PMC12891228; doi:10.3389/fneur.2026.1739214)

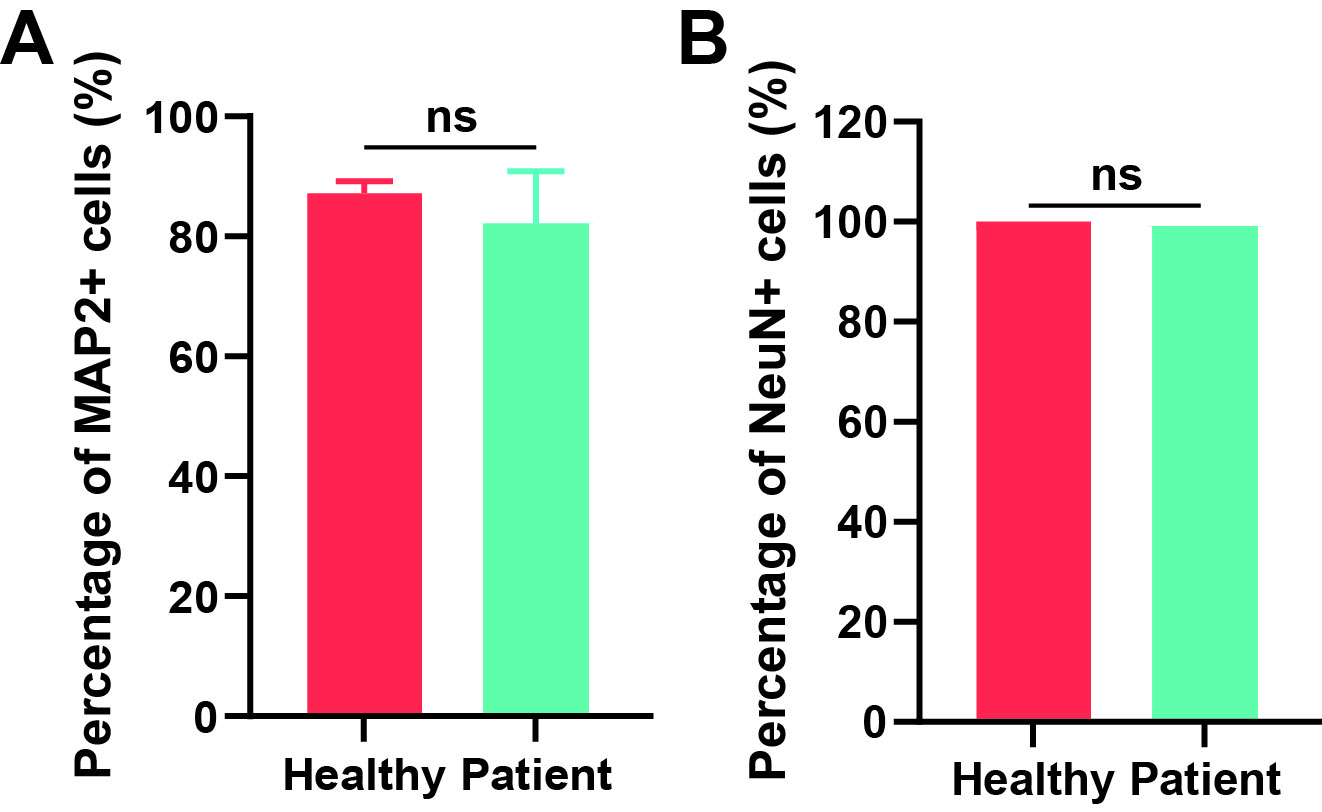

Supplement: Supplementary file 1 [file Image_1.jpeg]
